# Influence of acute kidney injury on short- and long-term outcomes in patients undergoing cardiac surgery: risk factors and prognostic value of a modified RIFLE classification

**DOI:** 10.1186/cc13159

**Published:** 2013-12-13

**Authors:** Juan C Lopez-Delgado, Francisco Esteve, Herminia Torrado, David Rodríguez-Castro, Maria L Carrio, Elisabet Farrero, Casimiro Javierre, Josep L Ventura, Rafael Manez

**Affiliations:** 1 Hospital Universitari de Bellvitge, Intensive Care Department, IDIBELL (Institut d’Investigació Biomèdica Bellvitge; Biomedical Investigation Institute of Bellvitge), C/Feixa Llarga s/n. 08907, L’Hospitalet de Llobregat, Barcelona, Spain; 2 Physiological Sciences II Department, Universitat de Barcelona, IDIBELL, Barcelona, Spain

## Abstract

**Introduction:**

The development of acute kidney injury (AKI) is associated with poor outcome. The modified RIFLE (risk, injury, failure, loss of kidney function, and end-stage renal failure) classification for AKI, which classifies patients with renal replacement therapy needs according to RIFLE failure class, improves the predictive value of AKI in patients undergoing cardiac surgery. Our aim was to assess risk factors for post-operative AKI and the impact of renal function on short- and long-term survival among all AKI subgroups using the modified RIFLE classification.

**Methods:**

We prospectively studied 2,940 consecutive cardiosurgical patients between January 2004 and July 2009. AKI was defined according to the modified RIFLE system. Pre-operative, operative and post-operative variables usually measured on and during admission, which included main outcomes, were recorded together with cardiac surgery scores and ICU scores. These data were evaluated for association with AKI and staging in the different RIFLE groups by means of multivariable analyses. Survival was analyzed via Kaplan-Meier and a risk-adjusted Cox proportional hazards regression model. A complete follow-up (mean 6.9 ± 4.3 years) was performed in 2,840 patients up to April 2013.

**Results:**

Of those patients studied, 14% (n = 409) were diagnosed with AKI. We identified one intra-operative (higher cardiopulmonary bypass time) and two post-operative (a longer need for vasoactive drugs and higher arterial lactate 24 hours after admission) predictors of AKI. The worst outcomes, including in-hospital mortality, were associated with the worst RIFLE class. Kaplan-Meier analysis showed survival of 74.9% in the RIFLE risk group, 42.9% in the RIFLE injury group and 22.3% in the RIFLE failure group (*P* <0.001). Classification at RIFLE injury (Hazard ratio (HR) = 2.347, 95% confidence interval (CI) 1.122 to 4.907, *P* = 0.023) and RIFLE failure (HR = 3.093, 95% CI 1.460 to 6.550, *P* = 0.003) were independent predictors for long-term patient mortality.

**Conclusions:**

AKI development after cardiac surgery is associated mainly with post-operative variables, which ultimately could lead to a worst RIFLE class. Staging at the RIFLE injury and RIFLE failure class is associated with higher short- and long-term mortality in our population.

## Introduction

The development of acute kidney injury (AKI) after adult cardiac surgery is associated with higher morbidity and mortality [1-3]. AKI develops in 1% to 30% of these patients, depending on the definition used for AKI [4], and leads to renal replacement therapy (RRT) in 1% to 5% [5]. Previous reports have studied risk factors associated with the occurrence of AKI, mainly focusing on factors measurable before surgery [6,7] or during the perioperative period [1]. However, postoperative management in the intensive care unit (ICU) could also be relevant for the occurrence of AKI after cardiac surgery. In addition, factors that are measurable postoperatively may indicate AKI development, suggesting appropriate strategies to prevent or limit AKI.

The RIFLE (Risk, Injury, Failure, Loss of kidney function, and End-stage renal failure) classification indicates AKI severity based on changes in serum creatinine (sCr) relative to the baseline condition, its association with short-term mortality after cardiac surgery having been validated previously [8-10]. Recently, a modification of the RIFLE classification by staging all patients with acute need for RRT in the failure class F showed an improvement of the predictive value for AKI in patients undergoing cardiac surgery, being superior to acute kidney injury network criteria (AKIN) if there is no correction of sCr for fluid balance, which leads to over-diagnosis of AKI [3]. In addition, data on long-term survival after AKI in these patients are scarce despite the need for such information.

The aim of this study was: (1) to identify the risk factors for AKI, especially regarding postoperative variables, and the predictive value for AKI of preoperative and postoperative scores; and (2) to evaluate the long-term mortality risk associated with a modified RIFLE classification after cardiac surgery in a large single-center cohort of patients with no history of chronic kidney disease, together with an evaluation of the variables that influence staging in the different RIFLE groups.

## Methods

This study was a retrospective study of prospectively collected data from 2,940 consecutive patients undergoing different types of cardiac surgery between January 2004 and July 2009 at our institution. We excluded those with preoperative renal failure requiring dialysis (*n* =24) or chronic kidney disease (CKD) (*n* = 144). Heart-transplant patients (*n* = 124) were also excluded due to the higher AKI rates reported in previous studies, which may lead to bias [2]. Definition of CKD was based on the Society of Thoracic Surgeons’ national cardiac surgery database definitions, which is defined as a serum creatinine value of 2.0 mg/dL (176.8 mmol · L-1) or greater.

The study was approved by the Institutional Ethics Committee of our hospital (Comité d’ Ètica i Assajos Clínics de Hospital Universitari de Bellvitge (CEIC); Ethics and Clinical Assays Committee of Hospital Universitari de Bellvitge). Informed consent was waived due to the observational nature of our study. The follow-up was performed using the Catalan Health Central Registry (*Registre Central de Persones Assegurades*, RCA). A complete follow-up was performed in 2,840 patients up to April 2013.

Data on and during ICU admission were extracted from the medical registry of each patient in real time using a standardized questionnaire and collected in a database for analysis purposes. Recent myocardial infarction (AMI) was defined as an AMI that required admission to the hospital during the last month before surgery or an AMI that did not allow discharge from the hospital before surgery. The other definitions used for this study were based on the Society of Thoracic Surgeons’ national cardiac surgery database definitions [11].

Preoperative data (demographic data, comorbidities and treatment before surgery), operative data and postoperative variables usually measured on and during admission, which included main outcomes, were recorded together with cardiac surgery scores (Parsonnet, European System for Cardiac Operative Risk Evaluation (EuroSCORE)) and ICU scores (Acute Physiology and Chronic Health Evaluation (APACHE) II and III, Simplified Acute Physiology Score (SAPS) II and III).

AKI was defined according to the RIFLE classification [8-10]. The baseline sCr is based on the preoperative analysis 24 h before surgery. Patients who met the RIFLE criteria for AKI were classified as “AKI”, whereas those who did not were classified as “no AKI”. Patients with AKI were stratified according to the RIFLE class; all patients with acute RRT were assigned to failure class F [3]. We measured the patients’ sCr at admission, 6 h, 12 h and 24 h postoperatively and a minimum of twice per day during their stay in the ICU based on our unit protocols.

The operations were performed by the same group of cardiac surgeons during the study period. Cardiac procedures were performed in all patients using median sternotomy, standard cardiopulmonary bypass (CPB) with moderate hypothermia (34°C) and antegrade cardioplegia. A mean aortic pressure of >60 mmHg was maintained during surgery. For revascularization, we used the internal thoracic artery (or bilateral if possible) and saphenous vein grafts. Bypass graft flow was assessed for each graft by Doppler transit time flowmetry. Protamine was administered to reverse heparin according to standard practice. For coronary artery bypass graft (CABG) surgery, aspirin was routinely administered within the first 6 h after surgery following the local protocol. In all patients the decisions regarding postoperative ICU management were made by the attending physician.

### Statistics

Statistical analysis was conducted using PASW statistics 13.0 (SPSS Inc., Chicago, IL, USA). Data are expressed as mean ± standard deviation. In order to evaluate differences regarding risk factors for AKI we analyzed differences between groups that were determined according to the presence of AKI after cardiac surgery. For comparisons between groups the Mann–Whitney *U* test was used or, when appropriate, the two-sample *t*-test. The *χ*^*2*^-test was used to evaluate categorical prognostic factors. A multivariate analysis was carried out using a stepwise logistic regression model to identify independent risk factors for AKI after cardiac surgery after adjusting for preoperative and postoperative scores. Receiver operating characteristic (ROC) curve analyses were applied to check the optimal cut-off values of the different scores for AKI diagnosis and to further evaluate the predictive power between them, considering the differences between the areas under the empirical ROC curves (AUC). ANOVA was used to compare differences in characteristics and outcome differences between different RIFLE class groups (*P* shown in tables) and subsequent *post hoc* tests (Bonferroni tests) were used to determine significant differences in the various pairwise comparisons (*P* shown in results). This was confirmed by means or a multivariate analysis after adjusting for preoperative and postoperative scores. In all cases, the Kolmogorov-Smirnov test was used to check the normal distribution of our population and to assess the goodness-of-fit of the final regression models. Survival analysis was carried out with the Kaplan-Meier estimator for the different RIFLE class groups. A proportional hazards Cox regression model was used to evaluate the effect of AKI and RIFLE class groups on survival. A two-tailed *P-*value <0.05 was considered statistically significant.

## Results

### Risk factors and scores prediction of AKI

The results of the univariate analysis of preoperative, intraoperative and postoperative data are shown in Tables 1 and 2. The 14% of patients (*n* = 409) who were diagnosed with AKI were older and more likely to have associated comorbid conditions and postoperative complications with a higher risk prediction for in-hospital mortality based on preoperative and postoperative scores than those without AKI.

**Table 1:** Univariate analysis of preoperative data associated with the presence of AKI after cardiac surgery

	**All patients**	**Non-AKI patients**	**AKI patients**	***P*-value**
	**(*n*** **= 2,940)**	**(*n*** **= 2,531; 86%)**	**(*n*** **= 409; 14%)**	
Sex (male)	64.0% (1,881)	64.4% (1,631)	61.1% (250)	0.20
Sex (female)	36.0% (1,059)	35.6% (900)	38.9% (159)	0.20
Age (years)	64.5 ± 11.6	64.0 ± 11.8	67.7 ± 9.8	**<0.001**
Hypertension	62.8% (1,846)	62.1% (1,570)	67.5% (276)	**0.03**
Dyslipidemia	50.5% (1,484)	51.4% (1,301)	44.9% (183)	**0.01**
Diabetes mellitus	8.2% (241)	7.9% (200)	10% (41)	0.14
BMI (kg · m^-2^)	28.1 ± 4.3	28.4 ± 4.3	27.9 ± 4.5	0.90
Peripheral vascular disease	8.9% (263)	7.9% (200)	15.4% (63)	**<0.001**
sCr before surgery (mmol · L^-1^)	95.8 ± 59.8	94.0 ± 60.0	101.0 ± 56.0	**0.045**
Previous stroke	5.6% (165)	5.2% (131)	8.3% (34)	**0.015**
COPD	12.0% (354)	11.6% (294)	14.7% (60)	0.08
Active smokers	15.5% (458)	15.8% (402)	13.7% (56)	0.29
Previous Atrial fibrillation	23.9% (703)	22.5% (569)	32.8% (134)	**0.045**
Previous myocardial infarction	15.4% (454)	16.0% (404)	12.2% (50)	0.055
Recent myocardial infarction	11.1% (325)	11.0% (278)	11.5% (47)	0.73
NYHA class III-IV	15.3% (450)	15.3% (389)	14.9% (61)	0.79
On B-Blockers	41.0% (1,204)	41.8% (1,057)	35.9% (147)	**0.026**
On statins	41.2% (1,212)	42.6% (1,078)	32.8% (134)	**<0.001**
On aspirin	44.4% (1,306)	45.7% (1,156)	36.7% (150)	**0.001**
On diuretics	47.6% (1,398)	46.0% (1,165)	57.0% (233)	**<0.001**
Hypertrophic cardiomyopathy	30.9% (910)	31.2% (790)	29.3% (120)	0.35
Dilated cardiomyopathy	20.4% (600)	20.1% (508)	22.5% (92)	0.34
LVEF (%)	60.2 ± 11.9	60.1 ± 11.8	60.3 ± 12.2	0.83
PAP (mmHg)	45.9 ± 15.7	45.3 ± 15.0	48.0 ± 17.0	**0.003**
Hemoglobin before surgery (g · dL^-1^)	12.9 ± 1.7	13.0 ± 1.6	12.4 ± 1.9	**<0.001**
Platelet count before surgery(1 · nL^-1^)	215 ± 68	217 ± 67	206 ± 76	**0.005**
EuroSCORE	5.9 ± 3.0	5.6 ± 2.7	7.7 ± 3.5	**0.015**
Parsonnet score	11.5 ± 7.3	10.9 ± 6.8	14.5 ± 9.3	**0.001**
Past cardiac surgery	9.4% (277)	8.4% (213)	15.6% (64)	**<0.001**

BMI, Body Mass Index; COPD, Chronic Obstructive Pulmonary Disease; NYHA, New York Heart Association classification; LVEF, Left ventricular ejection fraction; PAP, Pulmonary arterial pressure; sCr, serum creatinine;. Data are mean ± standard deviation or percentage.

Boldface data are statistically significant (*P* <0.05).

**Table 2:** Univariate analysis of intraoperative and postoperative data associated with the presence of AKI after cardiac surgery

	**All patients**	**Non-AKI patients**	**AKI patients**	***P-*** **value**
	**(*n*** **= 2,940)**	**(*n*** **= 2,531; 86%)**	**(*n*** **= 409; 14%)**	
**Intraoperative data**				
Isolated CABG	32.1% (945)	33.6% (851)	23.0% (94)	**0.005**
Isolated valve surgery	51.6% (1,518)	51.8% (1,311)	51.6% (207)	0.85
CABG + valve surgery	6.9% (203)	6.2% (158)	11.0% (45)	**<0.001**
Other cardiac surgery	9.4% (274)	8.4% (211)	15.4% (63)	**<0.001**
Emergent surgery	5.1% (149)	3.9% (99)	12.2% (50)	**<0.001**
Number of bypass	2.3 ±0.9	2.3 ± 0.8	2.2 ± 0.9	0.68
ACC time (minutes)	73 ± 29	72 ± 28	87 ± 37	**<0.001**
CPB time (minutes)	113 ± 41	109 ± 37	135 ± 55	**<0.001**
**Postoperative data**				
Ventilation time (hours)	50 ± 127	36 ± 96	139 ± 229	**<0.001**
PaO_2_/FiO_2_ ratio on admission	331 ± 98	334 ± 96	315 ± 106	**0.001**
PaO_2_/FiO_2_ ratio 12 h after admission	311 ± 89	316 ± 87	278 ± 93	**<0.001**
PaO_2_/FiO_2_ ratio 24 h after admission	307 ± 77	314 ± 72	270 ± 90	**<0.001**
Reintubation	1.1% (31)	0.8% (21)	2.5% (10)	**0.001**
Tracheostomy	1.3% (38)	0.8% (19)	4.7% (19)	**0.001**
Need of vasoactive drugs (hours)	103 ± 141	82 ± 110	195 ± 210	**<0.001**
LCOS	41.6% (1,223)	36.3% (920)	74.1% (303)	**<0.001**
PMI	11.8% (346)	10% (252)	23% (94)	**<0.001**
IABP support	7.8% (230)	6.1% (155)	18.3% (75)	**<0.001**
Atrial fibrilation	39.4% (1,158)	36.1% (913)	59.9% (245)	**<0.001**
AL on admission (mmol · L^-1^)	2.3 ± 1.4	2.2 ± 1.2	3.1 ± 2.3	**<0.001**
AL 24 h after admission (mmol · L^-1^)	1.9 ± 1.0	1.8 ± 0.7	2.6 ± 1.9	**<0.001**
sCr peak after surgery (mmol · L^-1^)	114.3 ± 80.8	99.0 ± 62.0	205.0 ± 113.0	**<0.001**
Albumin 48 h after surgery (g · L^-1^)	28.2 ± 3.7	28.4 ± 3.6	26.5 ± 4.1	**<0.001**
Hemorrhage-related re-exploration	3.5% (103)	2.6% (66)	9.0% (37)	**<0.001**
Pericardial tamponade	0.7% (22)	0.4% (11)	2.7% (11)	**<0.001**
Drainage loss first 12 h (mL)	393 ± 301	377 ± 275	496 ± 414	**<0.001**
Major bleeding	3.6% (109)	2.6% (66)	10.5% (43)	**<0.001**
Re-exploration	1.6% (48)	0.8% (21)	5.1% (27)	**<0.001**
Need for blood products (units)	1.6 ± 3.0	1.4 ± 2.5	3.3 ± 4.6	**<0.001**
Stroke	1.4% (42)	0.9% (24)	4.4% (18)	**<0.001**
Septicemia	6.6% (195)	4.0% (102)	22.7% (93)	**<0.001**
SAPS II	24.2 ± 9.6	22.8 ± 8.3	32.3 ± 12.8	**<0.001**
SAPS III	39.9 ± 10.4	38.6 ± 9.4	48.4 ± 12.3	**<0.001**
APACHE II	12.3 ± 4.6	11.6 ± 4.0	16.1 ± 6.3	**<0.001**
APACHE III	49.9 ± 18.5	47.0 ± 15.0	67.0 ± 24.0	**<0.001**
Mean Pre-ICU stay (days)	7.0 ± 13.0	6.7 ± 8.6	8.7 ± 28.8	0.15
Mean ICU stay (days)	7.5 ± 11.0	6.2 ± 7.7	15.2 ± 20.3	**<0.001**
Mean hospital stay (days)	24.6 ± 22.5	22.8 ± 15.7	36.1 ± 44.5	**<0.001**
In-hospital mortality	6.0% (177)	2.4% (60)	28.6% (117)	**<0.001**

ACC, Aortic cross clamping; AL, Arterial lactate; APACHE, Acute Physiology and Chronic Health Evaluation; CABG, Coronary artery bypass graft; CPB, Cardiopulmonary bypass; IABP, intra-aortic balloon pump; LCOS, Low Cardiac Output Syndrome; PaO_2_/FiO_2_, Arterial partial pressure of O_2_ and fraction of inspired oxygen ratio; PMI, Perioperative myocardial infarction; SAPS, Simplified Acute Physiology Score; sCr, serum creatinine. Data are mean ± standard deviation or percentage.

Boldface data are statistically significant (*P* <0.05).

In Table 3, we compare the results of the multivariate analysis of AKI based on different variable categories included in each analysis. We performed an adjustment for these scores in order to avoid the influence of severity of illness at the time of cardiac surgery and/or ICU admission. The preoperative data (older age, presence of peripheral vascular disease, higher pulmonary arterial pressure in preoperative echocardiography, and lower hemoglobin before surgery), intraoperative data (higher cardiopulmonary bypass (CPB) time and emergent surgery), and postoperative data (a longer need for vasoactive drugs and higher arterial lactate 24 h after admission) were associated with the occurrence of AKI when we analyzed these different variable categories separately. However, when assessing all data collected simultaneously, only postoperative variables and a higher CPB time were associated with the occurrence of AKI.

**Table 3:** Logistic regression model - dependent variable presence of AKI

	**Odds ratio (95% CI)**	***P-*** **value**
**Preoperative data**		
Age	1.038 (1.021 to 1.055)	**<0.001**
Presence of peripheral vascular disease	1.403 (0.991 to 1.987)	**0.003**
PAP (mmHg)	1.012 (1.002 to 1.022)	**0.020**
Hemoglobin before surgery (g · dL^-1^)	0.856 (0.783 to 0.936)	**0.001**
**Intraoperative data**		
CPB time (minutes)	1.013 (1.010 to 1.016)	**<0.001**
Emergent surgery	1.273 (1.168 to 1.444)	**<0.001**
**Postoperative data**		
Need of vasoactive drugs (hours)	1.005 (1.001 to 1.008)	**0.001**
AL 24 h after admission	1.530 (1.293 to 1.819)	**<0.001**
**All data**		
Need of vasoactive drugs (hours)	1.003 (1.002 to 1.004)	**<0.001**
AL 24 h after admission	1.810 (1.300 to 2.015)	**<0.001**
CPB time (minutes)	1.012 (1.002 to 1.028)	**0.025**

AL, Arterial lactate; CPB, Cardiopulmonary bypass; PAP, Pulmonary arterial pressure; sCr, Serum creatinine.

Boldface data are statistically significant (*P* <0.05).

When we assessed the ability of cardiac surgery and ICU scores to predict AKI (see Table 4), we found that cardiac surgery scores were poor predictors of AKI development whereas ICU scores were fair predictors based on the ROC curve.

**Table 4:** Comparison of AUC for ICU and cardiac surgery scores for AKI prediction

	**AUC ± SD% (95% CI)**	**Cut-off levels**	**Sensitivity**	**Specificity**	***P-*** **value**
APACHE II	71.0 ± 2.4 (66.4 to 75.6)	13.5	67.1%	64.7%	**<0.001**
APACHE III	75.8 ± 2.2 (71.4 to 80.1)	54.5	73.0%	67.1%	**<0.001**
SAPS II	72.3 ± 2.3 (67.7 to 76.9)	26.5	67.8%	65.7%	**<0.001**
SAPS III	72.0 ± 2.2 (67.6 to 76.3)	42.5	70.4%	61.9%	**<0.001**
EuroSCORE	67.6 ± 2.3 (63.0 to 72.2)	5.5	71.1%	53.8%	**<0.001**
Parsonnet	61.9 ± 2.5 (57.0 to 66.8)	11.5	61.8%	54.6%	**<0.001**

APACHE, Acute Physiology and Chronic Health Evaluation; AUC, Area under curve; EuroSCORE, European system for cardiac operative risk evaluation; SAPS, Simplified Acute Physiology Score.

Boldface data are statistically significant (*P* <0.05).

### Differences between RIFLE groups

The differences between RIFLE groups showed a comparable univariate association of the majority of outcome variables with worse outcome according to increased severity of AKI (see Tables 5 and 6). Preoperative variables showed lower hypertension rates in the RIFLE risk (RIFLE-R) group compared with the RIFLE failure (RIFLE-F) group and lower diabetes mellitus rates compared with the RIFLE injury (RIFLE-I) group. The RIFLE-F group suffered from higher CPB times compared with RIFLE-R (Bonferroni *post hoc P* <0.001) during cardiac surgery. Postoperative variables showed higher albumin levels 48 h after cardiac surgery in the RIFLE-R (*P* <0.001) and RIFLE-I (*P* = 0.019) groups when compared with RIFLE-F. The RIFLE-F and RIFLE-I groups showed a longer need for vasoactive drugs (*P* <0.001 in both groups) and higher in-hospital mortality rates (*P* = 0.001 and *P* = 0.003, respectively) when compared with the RIFLE-R group. Finally, the RIFLE-R group showed lower Low Cardiac Output Syndrome (LCOS) and septicemia rates compared with the RIFLE-F group. All these comparisons were confirmed later by means of the logistic regression model adjusted for risk prediction scores (see Table 7).

**Table 5:** Differences in preoperative data between AKI subgroups based on RIFLE classification

	**AKI patients**	**RIFLE risk**	**RIFLE injury**	**RIFLE failure**	***P-*** **value**
	**(*n*** **= 409)**	**(*n*** **= 226; 55.2%)**	**(*n*** **= 87; 21.3%)**	**(*n*** **= 96; 23.5%)**	
Sex (male)	61.1% (250)	58.8% (133)	57.5% (50)	69.8% (67)	0.12
Sex (female)	38.9% (159)	41.2% (93)	42.5% (37)	30.2% (29)	0.13
Age (years)	67.7 ± 9.8	67.3 ± 10.0	68.1 ± 10.4	68.1 ± 8.9	0.68
Hypertension	67.5% (276)	62.8% (142)	69.0% (60)	77.1% (74)	**0.042**
Dyslipidemia	44.9% (183)	39.4% (89)	46.0% (40)	56.3% (54)	**0.02**
Diabetes mellitus	10.0% (41)	7.5% (17)	17.2% (15)	9.4% (9)	**0.036**
BMI (kg · m^-2^)	27.9 ± 4.4	27.8 ± 4.3	28.2 ± 4.1	27.9 ± 5.3	0.77
Peripheral vascular disease	15.4% (63)	13.3% (30)	14.9% (13)	20.8% (20)	0.22
sCr before surgery (mmol · L^-1^)	101 ± 56	86 ± 31	91 ± 30	115 ± 48	**<0.001**
Previous stroke	8.3% (34)	7.5% (17)	10.3% (9)	8.3% (8)	0.72
COPD	14.7% (60)	14.2% (32)	17.2% (15)	13.5% (13)	0.74
Active smokers	13.7% (56)	10.2% (23)	19.5% (17)	16.6% (16)	0.43
Previous atrial fibrillation	32.8% (134)	31.4% (71)	31.0% (27)	37.5% (36)	0.61
Previous myocardial infarction	12.2% (50)	11.5% (26)	13.8% (12)	12.5% (12)	0.85
Recent myocardial infarction	11.5% (47)	8.8% (20)	14.9% (13)	14.6% (14)	0.17
NYHA class III-IV	14.9% (61)	15.1% (34)	14.9% (13)	14.6% (14)	0.82
On B-blockers	35.9% (147)	35.4% (80)	41.4% (36)	32.3% (31)	0.42
On statins	32.8% (134)	29.2% (66)	39.1% (34)	35.4% (34)	0.21
On aspirin	36.7% (150)	34.1% (77)	41.4% (36)	38.5% (37)	0.44
On diuretics	57.0% (233)	52.7% (119)	63.2% (55)	61.5% (59)	0.14
Hypertrophic cardiomyopathy	29.3% (120)	27.8% (63)	27.5% (24)	34.3% (33)	0.37
Dilated cardiomyopathy	22.5% (92)	20.8% (47)	25.3% (22)	23.9% (23)	0.65
LVEF (%)	60.0 ± 12.2	60.8 ± 11.7	58.7 ± 13.7	60.7 ± 11.8	0.41
PAP (mmHg)	48.0 ± 17.0	46.4 ± 16.9	51.9 ± 16.7	52.0 ± 16.9	0.07
Hemoglobin before surgery (g · dL^-1^)	12.4 ± 1.9	12.7 ± 1.8	12.2 ± 1.8	12.1 ± 2.1	**0.009**
Platelet count before surgery (1 · nL^-1^)	206 ± 76	206 ± 77	206 ± 75	205 ± 73	0.98
EuroSCORE	7.7 ± 3.5	7.1 ± 3.0	7.9 ± 3.6	8.8 ± 4.0	**0.028**
Parsonnet score	14.5 ± 9.3	13.1 ± 7.7	14.3 ± 7.8	18.5 ± 12.8	**<0.001**
Past cardiac surgery	15.6% (64)	17.3% (39)	11.5% (10)	15.6% (15)	0.45

AKI, Acute Kidney Injury; BMI, Body Mass Index; COPD, Chronic Obstructive Pulmonary Disease; LVEF, Left ventricular ejection fraction; NYHA, New York Heart Association classification; PAP, Pulmonary arterial pressure; sCr, serum creatinine. Data are mean ± standard deviation or percentage.

Boldface data are statistically significant (*P* <0.05).

**Table 6:** Differences in intraoperative and postoperative data between AKI subgroups based on RIFLE classification

	**AKI patients**	**RIFLE risk**	**RIFLE injury**	**RIFLE failure**	***P-*** **value**
	**(*n*** **= 409)**	**(*n*** **= 226; 55.2%)**	**(*n*** **= 87; 21.3%)**	**(*n*** **= 96; 23.5%)**	
**Intraoperative data**					
Isolated CABG	23% (94)	22.1% (50)	26.4% (23)	21.9% (21)	0.87
Isolated valve surgery	51.6% (207)	51.3% (116)	49.4% (43)	50.0% (48)	0.82
CABG + valve surgery	11% (45)	12.8% (29)	4.6% (4)	12.5% (12)	0.32
Other cardiac surgery	15.4% (63)	13.7% (31)	19.5% (17)	15.6% (15)	0.68
Emergent surgery	12.2% (50)	9.3% (21)	7% (8)	22.9% (22)	**0.004**
Number of bypass	2.29 ± 0.92	2.3 ± 0.9	2.5 ± 0.8	2.1 ± 1.0	0.21
ACC time (minutes)	87 ± 37	81 ± 32	96 ± 41	92 ± 39	**0.004**
CPB time (minutes)	135 ± 55	123 ± 44	147 ± 64	151 ± 62	**<0.001**
**Postoperative data**					
Ventilation time (hours)	139 ± 229	84 ± 176	204 ± 264	209 ± 268	**<0.001**
PaO_2_/FiO_2_ ratio on admission	315 ± 106	325 ± 105	320 ± 85	310 ± 115	**0.01**
PaO_2_/FiO_2_ ratio 12 h after admission	278 ± 93	300 ± 85	290 ± 105	270 ± 96	**0.003**
PaO_2_/FiO_2_ ratio 24 h after admission	270 ± 90	295 ± 78	259 ± 91	221 ± 94	**<0.001**
Reintubation	2.5% (10)	1.8% (4)	3.4% (3)	3.2% (3)	0.08
Tracheostomy	4.7% (19)	4.1% (9)	8.0% (7)	3.2% (3)	**0.01**
Need of vasoactive drugs (hours)	195 ± 210	137 ± 149	242 ± 207	267 ± 274	**<0.001**
LCOS	74.1% (303)	62.4% (141)	87.3% (76)	89.5% (86)	**<0.001**
PMI	23.0% (94)	14.6% (33)	28.7% (25)	37.5% (36)	**<0.001**
IABP support	18.3% (75)	15.9% (36)	16.0% (14)	26.1% (25)	**0.04**
Atrial fibrilation	59.9% (245)	52.2% (118)	64.4% (56)	74.0% (71)	**<0.001**
AL on admission (mmol · L^-1^)	3.1 ± 2.3	3.1 ± 2.2	2.9 ± 3.3	3.3 ±2.5	0.11
AL 24 h after admission (mmol · L^-1^)	2.6 ± 1.9	2.2 ±1.3	2.7 ± 1.7	3.3 ± 2.7	**<0.001**
sCr peak after surgery (mmol · L^-1^)	205 ± 113	143 ± 52	214 ± 72	342 ± 126	**<0.001**
Albumin 48 h after surgery (g · L^-1^)	26.5 ± 4.1	27.0 ± 3.4	26.0 ± 3.5	24.0 ± 5.0	**<0.001**
Hemorrhage-related re-exploration	9.0% (37)	5.3% (12)	13.8% (12)	13.5% (13)	**0.014**
Pericardial tamponade	2.7% (11)	2.2% (5)	2.3% (2)	4.2% (4)	0.59
Drainage loss first 12 h (mL)	496 ± 414	448 ± 368	523 ± 498	581 ± 422	**0.026**
Major bleeding	10.5% (43)	10.1% (23)	10.3% (9)	11.4% (11)	0.65
Re-exploration	5.1% (27)	4.4% (10)	4.6% (4)	13.5% (13)	**0.001**
Need for blood products (Units)	3.3 ± 4.6	2.6 ± 4.2	4.23 ± 4.9	4.3 ± 4.9	**0.001**
Stroke	4.4% (18)	3.1% (7)	3.4% (3)	8.3% (8)	0.09
Septicemia	22.7% (93)	13.2% (30)	27.5% (24)	40.6% (39)	**<0.001**
SAPS II	32.3 ± 12.8	27.7 ± 9.5	34.4 ± 13.0	40.9 ± 14.4	**<0.001**
SAPS III	48.4 ± 12.3	44.1 ± 9.9	49.2 ± 11.5	56.9 ± 13.2	**<0.001**
APACHE II	16.1 ± 6.3	13.7 ± 4.3	17.0 ± 6.8	20.9 ± 7.1	**<0.001**
APACHE III	67.0 ± 24.0	58.4 ± 17.4	69.6 ± 23.4	86.0 ± 27.5	**<0.001**
Mean Pre-ICU stay (days)	8.7 ± 28.8	6.2 ± 7.8	7.7 ± 9.3	15.7 ± 57.3	**0.023**
Mean ICU stay (days)	15.2 ± 20.3	12.0 ± 15.8	18.0 ± 22.8	20.3 ± 25.4	**0.001**
Mean hospital stay (days)	36.1 ± 44.5	31.5 ± 34.2	38.5 ± 32.0	44.6 ± 68.0	**0.046**
In-hospital mortality	28.6% (117)	10.6% (24)	42.5% (37)	58.3% (56)	**<0.001**

ACC, Aortic cross clamping; AL, Arterial lactate; APACHE, Acute Physiology and Chronic Health Evaluation; CABG, Coronary artery bypass graft; CPB, Cardiopulmonary bypass; IABP, Intra-aortic balloon pump; LCOS, Low Cardiac Output Syndrome; PaO_2_/FiO_2_, Arterial partial pressure of O_2_ and fraction of inspired oxygen ratio; PMI, Perioperative myocardial infarction; SAPS, Simplified Acute Physiology Score; sCr, Serum creatinine. Results are expressed as mean ± standard deviation or percentage.

Boldface data are statistically significant (*P* <0.05).

**Table 7:** Differences between RIFLE groups in a logistic regression model

	**Odds ratio (95% CI)**	***P-*** **value**
RIFLE risk vs RIFLE failure		
Hypertension	1.299 (1.098 to 1.916)	**0.034**
Cardiopulmonary bypass time (minutes)	1.014 (1.003 to 1.025)	**0.014**
Need of vasoactive drugs (hours)	1.003 (1.000 to 1.006)	**0.004**
Albumin 48 h after surgery (g · L^-1^)	0.858 (0.764 to 0.964)	**0.010**
Low cardiac output syndrome	1.144 (1.039 to 1.534)	**0.004**
Septicemia	1.078 (1.019 to 1.321)	**<0.001**
In-hospital mortality	1.856 (1.198 to 3.028)	**0.001**
RIFLE risk vs RIFLE injury		
Diabetes mellitus	1.323 (1.116 to 1.901)	**0.031**
Need of vasoactive drugs (hours)	1.002 (1.000 to 1.004)	**0.045**
In-hospital mortality	1.656 (1.360 to 2.980)	**0.003**
RIFLE injury vs RIFLE failure		
Albumin 48 h after surgery (g · L^-1^)	0.896 (0.828 to 0.969)	**0.006**

Boldface data are statistically significant (*P* <0.05).

### Mortality and survival analysis

A Cox proportional hazards model for patients’ in-hospital mortality demonstrated that staging at RIFLE-I (hazard ratio (HR) = 2.347, 95% confidence interval (CI) 1.122 to 4.907, *P* = 0.023) and RIFLE-F (HR = 3.093, 95% CI 1.460 to 6.550, *P* = 0.003) were independent predictors for patient mortality. Other factors associated with an increased risk of death included older age (HR = 1.080, 95% CI 1.036 to 1.126, *P* <0.001), diabetes mellitus (HR = 1.376, 95% CI 1.178 to 1.795, *P* = 0.01), longer time on vasoactive drugs (HR = 1.003, 95% CI 1.001 to 1.004, *P* <0.001) and suffering a stroke after cardiac surgery (HR = 1.130, 95% CI 1.045 to 1.376, *P* <0.001).

**Figure 1:**
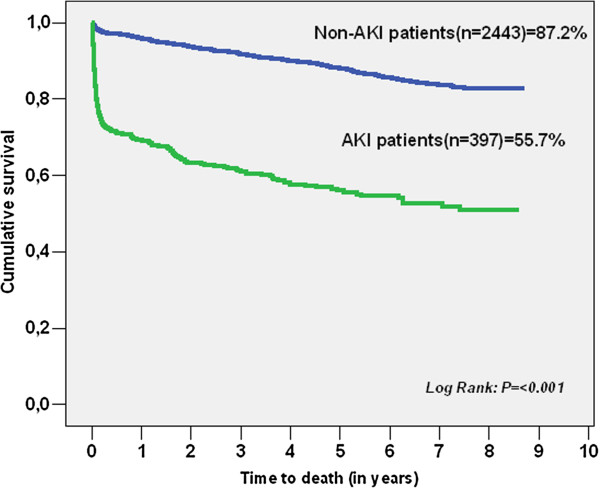
**Kaplan-Meier survival curves for the presence of AKI.**

**Figure 2:**
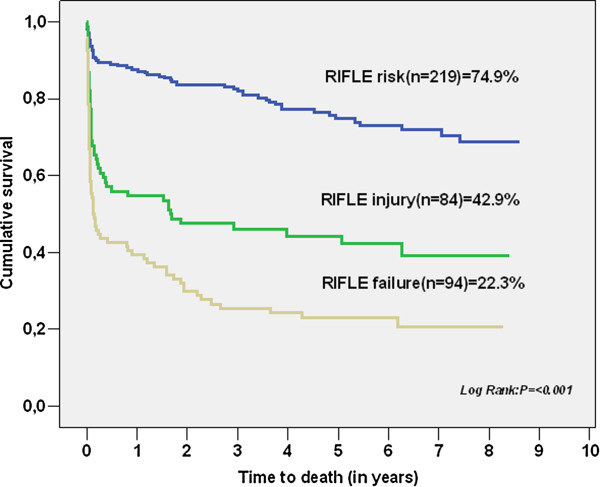
Kaplan-Meier survival curves for the different AKI groups.

**Figure 3:**
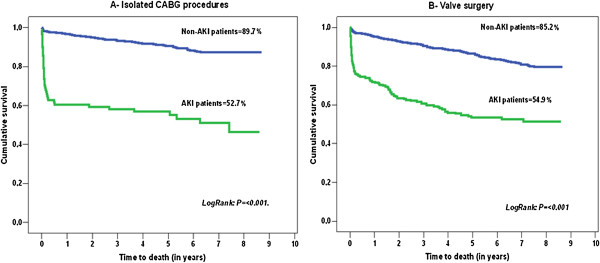
Kaplan-Meier survival curves for AKI patients stratified by individual surgery type (A and B).

We performed a complete follow-up in order to evaluate long-term mortality in 2,840 patients. Mean follow-up was 6.9 ± 4.3 years. Kaplan-Meier plots, shown in Figures 1 and 2, illustrated that patients with AKI and a higher RIFLE class had worse long-term survival over the follow-up period (see also Table 8). The long-term survival was similar regardless of type of surgery, as shown in Figure 3A, B. A Cox proportional hazards model of patient mortality demonstrated that AKI in isolated coronary artery bypass graft (CABG) procedures (HR = 3.706, 95% CI 2.012 to 6.875, *P* <0.001) and valve surgery procedures (HR = 2.713, 95% CI 1.980 to 5.250, *P* <0.001) was an independent predictor of mortality in these surgical groups. We observed a long-term global mortality of 11.74% (*n* = 313/2,665), after excluding patients who died in-hospital and those who survived but in whom follow-up could not be performed. In addition, in the long-term scenario mortality was 10.6% in non-AKI patients (*n* = 253/2,384), 21.4% (*n* = 60/281) in AKI patients, 15.9% (*n* = 31/195) in RIFLE-R, 25% (*n* = 12/48) in RIFLE-I, and 44.7% (*n* = 17/38) in RIFLE-F (*P* <0.001).

**Table 8:** Survival rates during follow-up for the different AKI groups

**RIFLE class**	**1-year**	**2-years**	**3-years**	**4-years**	**5-years**	**6-years**	**7-years**	**8-years**	**9-years**
Risk	88%	84%	82%	77%	75%	73%	72%	68%	68%
Injury	55%	48%	46%	45%	45%	42%	39%	39%	39%
Failure	39%	30%	25%	24%	23%	23%	20%	20%	20%

## Discussion

This study shows the key importance of postoperative factors, which can be easily monitored, for predicting the occurrence of AKI after cardiac surgery. Thus, a prompt intervention in the postoperative management in the ICU, especially avoiding additional renal insults and optimizing volume status, may help to some extent to prevent a higher progression of perioperative AKI, and the occurrence of the worst outcomes, including in-hospital mortality, is associated with the worst RIFLE class. We also demonstrated that scoring systems based only on variables known preoperatively, such as the Parsonnet and EuroSCORE, which have been proposed for the assessment of AKI developing after adult cardiac surgery [12], are worse predictors than ICU scores, which mainly included variables known postoperatively. Finally, a modified RIFLE classification is associated with long-term mortality, especially when staging within the RIFLE-I and RIFLE-F groups.

Although sCr is not always a perfect surrogate of renal function, it continues to be a basic measurement for the classification and diagnosis of AKI [8]. A ≥10% reduction in the sCr level may predict significantly lower AKI risk, whereas a ≥10% increase may predict significantly higher AKI risk compared with the reference category, reflecting the fact that minimal changes in sCr can increase mortality after cardiac surgery [4,13]. Novel biomarkers, such as neutrophil gelatinase-associated lipocalin and cystatin C, have been correlated with the duration and severity of AKI and the duration of ICU stay after adult cardiac surgery, and have been identified as independent predictors of AKI, being superior to conventional biomarkers [14]. However, due to its availability and widespread use, sCr continues to be a more valuable and accepted tool for AKI diagnosis worldwide.

Hyperlactatemia in the ICU is associated with increased mortality, being more frequent when renal failure is present [15]. It predicts postoperative mortality after cardiac surgery with a maximum lactate threshold of ≥4.4 mmol · L^-1^ in the first 10 h after operation [16]. During CPB the kidneys may suffer from an imbalance between oxygen supply and oxygen needs, resulting in inadequate oxygen delivery that is associated with lactate production [17]. The duration of CPB, which is a surrogate of the complexity of the procedure or of unexpected intraoperative problems, and its related variables, such as pressures and flows, have also been associated with AKI [1,18,19]. This may explain why higher arterial lactate values, which are ultimately a surrogate marker of tissue hypoperfusion, and a longer CPB duration, were associated with the occurrence of AKI. In addition, oxygen delivery depends on an appropriate hemoglobin level [17], being consistent with our finding that lower hemoglobin before surgery was associated with AKI. Previous studies that found preoperative anemia, hemodilution and perioperative red blood cell transfusions to be associated with AKI are also consistent with this finding [19,20]. As a consequence, intraoperative avoidance of the extremes of anemia, especially during CPB, and avoidance of transfusion in patients with hemoglobin levels >8 g · dL^-1^, may be helpful strategies in order to decrease AKI in patients undergoing cardiac surgery [21,22].

Despite the relationship between heart failure and renal insufficiency, even in the acute scenario [23], there is a lack of studies associating heart failure variables and/or related variables with AKI after cardiac surgery [24]. The postoperative use of norepinephrine in postoperative cardiac surgery patients and the postoperative use of vasoactive drugs in those with sCr <60 mL · minute^-1^ · 1.73 m^-2^ has been associated with AKI [19]. We hypothesized that a longer requirement for vasoactive drugs, even with a higher RIFLE class, is a variable concerning the perioperative drug management of patients, and could be a surrogate marker of unresolved postoperative cardiac or vascular dysfunction.

We confirmed the association of worse outcomes, including in-hospital mortality, with a worse RIFLE class, which may ultimately contribute to AKI. Limited CPB duration and adequate cardiac output are of key importance in order to avoid AKI development [25]. Hypoalbuminemia also increased the risk for infection in cardiac surgery patients, which itself is an important risk factor for mortality after cardiac surgery [26]. Sepsis can induce cardiac dysfunction *per se*[27], being associated with AKI and mortality after cardiac surgery [28,29]. As a result, our findings are concordant with the literature in relation to the associated AKI factors described above.

The RIFLE classification provides a useful tool for identifying patients with AKI after cardiac surgery and as a consequence those at risk of death, even in the long-term scenario [1-4], being superior to the classical postoperative renal failure definition in identifying such patients [11]. The present report is the first detailing the important association between long-term mortality after cardiac surgery and RIFLE-I and RIFLE-F classes in a large, single-center cohort, defined by a modified RIFLE classification [3]. We have also shown that AKI is an independent predictor of outcome regardless of type of surgery, being more important in isolated CABG procedures, as previously reported, but with greater influence over valve surgery compared with other studies [2]. Peripheral vascular disease leads to endothelial dysfunction, which is associated with renal insufficiency and contributes to cardiovascular mortality [30]. We hypothesized that a higher influence of arteriosclerosis in CABG patients is also associated with peripheral vascular disease and with vascular damage in renal vessels, which ultimately predisposes to AKI.

Renal blood flow and clearance function can remain impaired for a prolonged period of time after an episode of AKI, despite apparent normalization of sCr [31]. Indeed, several studies have indicated that there is ongoing progressive damage after AKI that results in a decrease in the capillary density of peritubular capillaries, a process known as “rarefaction” that can be linked to the development of chronic kidney disease, often with a delayed increase in sCr [32]. We can only hypothesize that the development of chronic kidney disease is one of the potential mechanisms that exposes these patients to increased cardiovascular morbidity and mortality [33]. Although comparisons among other studies remain difficult due to the different definitions and incidence of AKI, our survival rates for both AKI and non-AKI groups are comparable with other studies [2].

Our study presents certain limitations. The most important is that it was a single-center observational study. Unfortunately, we were not able to collect information on the cause of death and progression of kidney disease either. Among the strengths of this study are the large sample size, the prospective entry of all data elements into the database and the use of the finest statistical models together with systematic risk assessment using preoperative and postoperative scores, which are not shown in contemporary studies, even since the widespread use and importance of risk score stratification during the last decades. Furthermore, this investigation was conducted at a large tertiary referral hospital with a high level of complexity and all types of surgery, and all patients underwent surgery with CPB.

## Conclusions

In summary, the cause of AKI in the postoperative period, which is usually multifactorial, could be associated to a large extent with postoperative variables. In most cases, such variables lead to worse RIFLE staging when AKI occurs. The occurrence of AKI, especially staging at the RIFLE-I and RIFLE-F class, is associated with higher long-term mortality in our population.

The identification of postoperative AKI predictors could be of great clinical value, suggesting management changes that could prevent or reduce the impact of AKI itself and guiding ICUs in allocating resources for postoperative care before more severe complications occur. In addition, on the basis of this and previous studies [2,31-33], we suggest that patients suffering AKI after cardiac surgery should be closely followed in order to detect progressive renal damage beyond the acute episode, despite apparent normalization of sCr.

## Key messages

• The occurrence of AKI in the postoperative period of cardiac surgery continues to be a crucial factor which influences the outcome these patients, even from the long-term perspective.

• AKI development after cardiac surgery is associated with postoperative variables, which ultimately could lead to a worse RIFLE class.

• Staging at the RIFLE injury and RIFLE failure class is associated with higher short- and long-term mortality in our population.

• The identification of postoperative AKI predictors could help clinicians in order to prevent the impact of AKI itself and guiding ICUs in allocating resources for postoperative care.

## Abbreviations

AKI: Acute kidney injury; AKIN: Acute kidney injury network criteria; AMI: Acute myocardial infarction; APACHE: Acute Physiology and Chronic Health Evaluation; CABG: Coronary artery bypass graft; CKD: Chronic kidney disease; CPB: Cardiopulmonary bypass; EuroSCORE: European System for Cardiac Operative Risk Evaluation; ICU: Intensive care unit; LCOS: Low cardiac output syndrome; RIFLE: Risk, injury, failure, loss of kidney function, and end-stage renal failure; RRT: Renal replacement therapy; SAPS: Simplified acute physiology score; sCr: serum creatinine.

## Competing interests

There is no funding support or conflicts of interest for the present paper.

## Authors’ contributions

JCLD was involved in the conception and design of the research, and performed statistical analysis and wrote the paper. FE performed statistical analysis and wrote the paper. HT was involved in the coordination and the acquisition of data. DRC contributed to the acquisition of data, especially in terms of follow-up. MLC and EF contributed to the design of the research and acquisition of data. CJ performed statistical analysis and interpretation of data. JLV was involved in the conception, design of the research and interpretation of data. RM was involved in the design of the research and supervised the writing of the present manuscript. All authors read and approved the final version of this manuscript.
